# Assessing health care in Canada's North: what can we learn from national and regional surveys?

**DOI:** 10.3402/ijch.v74.28436

**Published:** 2015-07-24

**Authors:** T. Kue Young, Carmina Ng, Susan Chatwood

**Affiliations:** 1 School of Public Health, University of Alberta, Edmonton, AB, Canada; 2 Institute of Medical Science, University of Toronto, Toronto, ON, Canada; 3 Institute for Circumpolar Health Research, Yellowknife, NT, Canada; 4 Dalla Lana School of Public Health, University of Toronto, Toronto, ON, Canada

**Keywords:** surveys, Canada, health services, Aboriginal people

## Abstract

**Background:**

Health surveys are a rich source of information on a variety of health issues, including health care.

**Objectives:**

This article compares various national and regional surveys in terms of their geographical coverage with respect to the Canadian North, especially their Aboriginal population, and the comparability of the survey contents relating to health care.

**Methods:**

Three surveys were selected as providing some information on health care, with separate estimates for the North and its Aboriginal populations. They are the Canadian Community Health Survey (CCHS), Aboriginal Peoples Survey (APS) and the First Nations Regional Health Survey (RHS).

**Results:**

Different surveys focus on different categories of Aboriginal people, and no single survey has covered all categories of Aboriginal people in the North consistently. RHS is targeted at the on-reserve First Nations population only. APS and CCHS sample the off-reserve First Nations population as well as Métis and Inuit. To achieve adequate sample size for North–South comparisons and comparisons among Aboriginal groups within the North, several cycles of the biennial/annual CCHS can be merged, producing a large data set with consistent coverage of topics using comparable questions. The content areas of the 3 surveys can be broadly categorized as health status, health determinants and health care. Substantial variation exists across surveys in the domains covered. There are also changes over time in terms of definitions, questions and even basic concepts. The available health care content of the 3 surveys focus on access to different types of health services, contact with different categories of health professionals, unmet health needs and the use of preventive services. Many important dimensions of health care are not covered. Not all these basic indicators are available for the North or its Aboriginal populations.

**Conclusions:**

A comprehensive survey of health care in the North with sufficient sample size to provide reliable estimates for its subpopulations – urban and remote, Aboriginal and non-Aboriginal, and First Nations, Inuit and Métis – would provide useful information to decision-makers and service providers. Analytical studies can also be conducted to investigate the correlations and interactions among health status, health determinants and health care and assess whether such relationships differ among the different population groups.

Health care is a major concern of residents of Canada's northern territories. Among the many health care challenges are a scattered and remote population, harsh environmental conditions and a thinly deployed health workforce (1,2). Despite per capita health care expenditures that are among the highest in the world, health outcomes continue to lag behind the rest of Canada, and health disparities between the Aboriginal and non-Aboriginal population within the North continue to persist (3–5). Evidence-based solutions are needed by those who use, those who provide, those who run, those who plan and those who fund health services.

One approach to understanding and investigating health care in the North is through surveys. However, conducting stand-alone surveys that are focused exclusively on health care in the North is resource intensive and time consuming.

## Objectives

This article explores the extent to which health care issues in northern Canada can be studied through secondary analyses of existing national and regional health and social surveys, as well as identifying potential areas for research.

## Methods

For the purpose of this scan, the North only includes the 3 territories of Yukon, Northwest Territories (NWT), and Nunavut.

We consulted extensively the various user guides, data dictionaries and other documentation of these surveys, and reviewed published studies that used these data sources. Some descriptive data obtained from published reports and data tables are presented to highlight certain issues and identify existing gaps, but no new analyses of microdata files were conducted.

## Health surveys in Canada

Many health surveys have been conducted in Canada over the years, at the national, provincial/territorial, regional and community levels. Some specifically exclude the northern territories. Some include First Nations, Inuit and Métis, while others are restricted only to one or more groups. Some are targeted at the on-reserve population, while others cover only the off-reserve population. Data collection methods differ among the surveys – while most involved only interviews (face-to-face or via telephone), a few also included clinical and laboratory measurements of participants.

We have identified the following surveys which contain some questions on health care, especially access and barriers, visits to health professionals and use of preventive health services.

### National Population Health Survey

The National Population Health Survey (NPHS) conducted by Statistics Canada was originally composed of 3 components: the Households, the Health Institutions and the North components (6). The Household component started in 1994/1995 and is conducted every 2 years. In each household, some limited information is collected from all household members and one person, aged 12 years and over, in each household was randomly selected for a more in-depth interview.

The first 3 cycles (1994/95, 1996/97 and 1998/99) were both cross-sectional and longitudinal. Beginning in Cycle 4 (2000/2001), the survey became strictly longitudinal.

First Nation reserves were excluded from all NPHS cycles. The North was not included in the longitudinal survey after Cycle 3. The NPHS will therefore not be discussed further.

### Canadian Community Health Survey

The Canadian Community Health Survey (CCHS), also conducted by Statistics Canada, is a cross-sectional survey of Canadians aged 12 and above with sample size large enough for comparisons at the health region level (7). The territories are included, with coverage of 92% of the population of Yukon, 96% of the NWT and 71% of Nunavut, where only the 10 largest communities were sampled. However, with the 2013 cycle, the coverage in Nunavut was increased to 92%. First Nations reserves and the predominantly Inuit region of Nunavik and James Bay Cree Territory in Québec are not included.

The first CCHS (Cycle 1.1) was conducted in 2000/01, followed by Cycle 2.1 (2003) and Cycle 3.1 (2005). There were also special focus content surveys – Cycle 1.2 (2002) on mental health, which was repeated in 2012, and Cycle 2.2 (2004) on nutrition, repeated in 2015. First Nations reserves and the North are excluded from these special surveys. Beginning with Cycle 4.1 (2007), CCHS became annual rather than biennial. The numbering system was discontinued after 4.1 and the cycles are referred to only by the year. In this article, all CCHS cycles will be referred to by the year.

The CCHS is composed of a *common* content and an *optional* content. Individual provinces and territories can choose to opt in or out of a variety of optional modules, over and above the core variables that are subscribed to by all jurisdictions. This is to enable provincial/territorial/regional health authorities to focus on issues that are of particular significance to them. Since 2007, a *rapid response* content has been added, which is offered to special-interest groups on a cost-recovery basis to obtain national estimates of specific health issues.

Post-2007, there are 2 types of common content modules/variables: the “annual” (called “core” in 2007 and 2008), which consists of questions asked in every year; the “one-year” questions, which are asked during 1 year, and repeated every second or fourth year; and the “two-year” questions, which are asked for 2 consecutive years and skipped the next 2 years. The 1- and 2-year modules/variables were called “theme” in 2007 and 2008.

All respondents in all regions answer the common content questions. All respondents in the opted regions answer the optional content questions. To add to the confusion, in certain years, there are also “subsamples,” which are subsets of respondents in participating regions. These are smaller in size, but just large enough to generate reliable national or provincial/territorial estimates. Throughout the years, some modules/variables move between these categories.

With the exception of CCHS Nutrition (in 2004 and 2015) and a subsample of CCHS 2005 where height and weight were actually measured rather than self-reported, CCHS is an exclusively interview-based survey.

### Aboriginal Peoples Survey

Although not exclusively a health survey, the Aboriginal Peoples Survey (APS) has always contained a variety of health-related variables. The APS was conducted by Statistics Canada as a post-censal survey after the censuses of 1991, 2001 and 2006. It included interviews only. There are Arctic and Métis supplements for 2001 and 2006. The Arctic supplements shared common questions with the multinational circumpolar Survey of Living Conditions in the Arctic (SLiCA) (8). While the long-form census was eliminated from the 2011 Census and replaced by the National Household Survey (NHS), the NHS did form the basis for APS 2012. An extensive Health Supplement was introduced with APS 2012.

APS 1991 included Métis, Inuit and both on- and off-reserve First Nations populations. The coverage of the on-reserve population was poor in APS 2001. Only 15% of the reserves that participated in the 2001 census participated in APS 2001. The 2006 and 2012 APS continued the practice of sampling the Métis and Inuit, but only the off-reserve First Nations populations. APS 2012 further excluded First Nations communities and the few official reserves in Yukon and the NWT. The First Nations population sampled was from among those living in cities, towns and unorganized settlements in the territories.

APS 2001 covered those aged 0–14, and 15 and above. The age ranges were changed to 6–14 and 15+ in APS 2006 and APS 2012. A companion Aboriginal Children Survey (ACS) was conducted in 2006 to survey the 0–5 age group no longer covered by APS 2006. There was, however, no ACS 2012.

### First Nations Regional Health Survey

The First Nations Regional Health Survey (RHS) is the only First Nations-governed and administered national health survey in Canada. It is intended to be longitudinal. It is an interview-only survey. The RHS is governed and coordinated nationally by the First Nations Information Governance Committee, with representation from regional First Nations organizations.

- RHS 1997: sampled 9,870 on-reserve First Nations adults from 9 provinces, with 517 Inuit from 5 communities in Labrador, the only time Inuit participated in RHS.
- RHS Phase 1 (2002/03): surveyed 22,602 adults, youths, and children from 238 First Nations communities in all provinces, Yukon and NWT.
- RHS Phase 2 (2008/10): in total 21,757 participants from 216 First Nations communities in all provinces, Yukon and NWT were surveyed.
- RHS Phase 3 was conducted in 2013/14 and analyses are underway

Regions have the option to add to the national core questionnaire: NWT in 2002/03 expanded on hunting, trapping and traditional activities; mental health; community life and wellness in the adult survey, whereas Yukon did not add questions. For 2008/09, Yukon focused on cultural activities, vitamin supplements and addiction treatment.

### Health Services Access Survey

This survey was conducted by Statistics Canada in 2001 with the specific focus on access to first contact health services and waiting time for key diagnostic and treatment services. Neither reserves nor the territories were included. In 2003, Health Services Access Survey (HSAS) was amalgamated with CCHS (9). The modules ACC (access to health care services) and WTM (waiting time) were administered as subsamples in CCHS 2005, 2007, 2009 and 2011, and as a rapid response content in 2013. In 2005, the 3 territories were included for the first and only time. CANSIM Table 105-3024 provides summary data from the ACC and WTM modules.

### Canadian Survey of Experiences with Primary Care

This survey was co-sponsored by the Canadian Institute for Health Information (CIHI) and the Health Council of Canada and was conducted as a subsample of CCHS (10). A nationally representative sample of individuals aged 18 and over was surveyed during 2007, with a sample size of 2,194. While the territories were not excluded, there were only 5 respondents. This could be considered a pilot for the larger survey in 2008, with a national sample size of 11,582, of whom only 15 were from the territories. An analysis of Canadian Survey of Experiences with Primary Care (CSE-PHC) focused on individuals with 1 or more of 5 chronic diseases and reported on their use of, access to and experiences with primary health care services and support for self-management of chronic diseases (11). The survey needs to be repeated in the territories with sufficient sample size to provide reliable estimates specific to the North.

### Other surveys

Other important health surveys have been conducted since 2000, for example, the Canadian Health Measures Survey (CHMS) (12) and the Inuit Health Survey (13), but these contain no health care questions. CHMS did not establish any sampling sites in the 3 northern territories.

Canada participates in international surveys of health care providers and patients, such as those organized by the Commonwealth Fund (14), but these do not include the northern territories.

In this report, we focus on the older cohorts of CCHS (12+), APS (15+) and RHS (18+) conducted since 2000.

## Coverage of target populations

Table I summarizes the age range, inclusion/exclusion of provinces and territories, and the separation of Aboriginal respondents into First Nations (on- and off-reserve), Métis and Inuit in selected surveys since 2000.

**Table I:** Coverage of target population in selected surveys

				Aboriginal population					
				
				First Nations				Location	
							
Survey	Cycle	Age range	aNon-Ab	On-reserve	Off-reserve	Métis	Inuit	South	North
CCHS	2000/01	12+							[1]
	2003	12+				[2]			[1]
	2005 and later	12+							[1]
APS	2001	0–14, 15+		[3]					[4]
	2006	6–14, 15+							[4]
	2012	6–14, 15+							[5]
RHS	2002/03	0–12, 12–17, 18+		[6]					[4]
	2008/10	0–12, 12–17, 18+		[6]					[4]

a “Non-Ab” refers to the non-Aboriginal population. “South” refers to the 10 provinces and “North” to the 3 territories.

[1] Covers only 92% of population in Yukon, 96% in NWT, and 71% in Nunavut (the 10 largest communities). With the 2013 cycle, the coverage has been increased to 92% in Nunavut.

[2] Only a combined “Aboriginal” category.

[3] Poor response rate.

[4] First Nation communities in Yukon and Northwest Territories covered.

[5] First Nation communities in Yukon and Northwest Territories not covered.

[6] Excluding James Bay Cree and Labrador Innu.

Surveys conducted by Statistics Canada generally do not cover First Nations reserves, leaving the RHS the only source of data on this segment of the Aboriginal population. Excluded from the table are surveys conducted in single or clusters of communities.

It is also clear that no single survey has covered all categories of Aboriginal people in the North consistently since 2000. When interpreting and comparing studies reporting estimates of health measures among northern Aboriginal people, close attention needs to be paid to the source survey used.

## Definition of Aboriginal People

There is a lack of consistency in the definition of an Aboriginal person in surveys. Over the decades, the approach used by Statistics Canada and the surveys it has conducted has changed (15). Basically, it has used 2 concepts, that of “identity” (i.e. Does the respondent consider himself/herself to be an Aboriginal person?) and “ancestry” or “origins” (i.e. Does the respondent have an ancestor who was an Aboriginal person?). The “identity-population” and “ancestry-population” do not always coincide. Among First Nations people, there is the additional issue of whether the person is “status” or “non-status,” and “on-reserve” or “off-reserve.” Only the ancestry question was used in CCHS 2000/01 and 2003. The identity question was added in CCHS 2005 and both were used in subsequent cycles. Both ancestry and identity questions were used in APS 2001 and 2006, but the ancestry-only population was excluded from APS 2012.

In a study that merged CCHS 2009 and 2010, it was found that, of participants who identified themselves as Aboriginal, only 63% reported having an Aboriginal ancestor; of those who claimed Aboriginal ancestry, only 57% identified themselves as Aboriginal (16). The lack of concordance differed according to whether the individual was First Nation, Métis or Inuit. The different methods of enumerating the Aboriginal population did not significantly affect the prevalence estimates of 3 common chronic conditions (diabetes, arthritis and hypertension). The impact on estimating other variables such as health care remains to be investigated.

In this article, unless otherwise specified, the Aboriginal population refers to the Aboriginal *identity* population.

## Sampling and data collection

Before 2007, CCHS data were collected over a 12-month period every 2 years, resulting in a sample size of about 130,000. In 2007 and subsequent years, data were collected in six 2-month periods each year from about 65,000 respondents, resulting in the same sample size over a 2-year period. For the provinces, the sample collected during each 2-month period is representative of the provincial population. For the territories, only the 12-month sample can be considered representative of the territorial population.

CCHS is a complex, multistaged, stratified cluster survey. It sampled participants using 3 sampling frames – an area frame, a list frame and a random digit dialling (RDD) frame. The area frame produces a sample that is representative of the geographical, urban/rural and socio-economic characteristics of the population. The list frame is based on listing of numbers in telephone directories. The RDD frame is derived from banks of telephone numbers after eliminating those that are non-residential or non-working. The penetration rate of telephones in northern Canadian homes is not known, as the North (and also First Nations reserves) are excluded from Statistics Canada's Annual Residential Telephone Service Survey. The lack of home phones could be a potential source of bias.

Nunavut used the area frame exclusively, while a small number of respondents living in the territorial capitals of Yukon and NWT were selected from RDD. In the North, the area frame consists of 5 strata in the Yukon, 10 in NWT and 6 or 7 in Nunavut. Each stratum may be a single large community or a group of smaller communities. For the latter, one community is randomly selected. The slightly different sampling in the territories affected the determination of sampling weights. Beginning in 2008, weighting for the territories took into consideration the composition of the population in terms of Aboriginal and non-Aboriginal, and those living in the capitals and in other communities. This change may affect comparability of estimates with previous years.

From the beginning, CCHS used computer-assisted interviewing (CAI), with some involving in-person interviews (CAPI) and others using the telephone (CATI). The proportions of these vary over the years.

As a post-censal survey, APS selects its subjects from those who have earlier answered several questions in the census indicating that they were Aboriginal people (defined in several different ways). There are important differences between APS 2012 and earlier cycles. In the 2001 and 2006 censuses, questions on Aboriginal identity and ancestry were included in the “long-form” administered only to about 20% of the Canadian population. Legally both the short-form (containing only a few demographic questions) and long-form censuses are compulsory under the *Statistics Act*, although since the early 1990s, many First Nations communities have not participated in the census. In 2011, the long-form census was replaced by the NHS, which is itself a complex 2-stage survey, but a voluntary one (17). APS 2012, thus, adds a third phase to the sampling. APS 2012 also has a special focus on education, and there are 4 strata based on school attendance and completion status.

Prior to 2012, APS used a paper questionnaire, administered both in person and over the telephone. APS 2012 introduced CAI, both CAPI and CATI.

Sampling in the RHS is relatively simple when compared to CCHS and APS, as it focuses solely on First Nations communities, including reserves. When the RHS was first launched in 1997, there was considerable regional autonomy in sampling, resulting in a mish-mash of regional data sets, which were combined into a national core data set with doubtful validity. However, these problems had been resolved in subsequent cycles. In fact, the RHS governance body has re-started the clock, now calling the 1997 cycle a “pilot,” and the 2002/03 phase 1 of a longitudinal cohort. The Harvard Project on American Indian Economic Development was contracted to evaluate the design, implementation, data management, analysis and dissemination of RHS 2002/03. The overall review was very positive, attesting to the quality of the survey overall (18). RHS 2008/10 similarly underwent external scrutiny by a team from the Johns Hopkins School of Public Health; however, that report is not available publicly.

CAPI was used in 90% of RHS 2002/03 respondents, administered by field workers. CAPI was used in RHS 2008/10, self-administered by adults and youths and administered by proxy for children.

## Sample size considerations

In general, Aboriginal-focused surveys such as APS and RHS are targeted at the Aboriginal population and have sufficient sample size for most statistical analyses. They are also adequate for the purpose of generating prevalence estimates for broad age–sex and regional subgroups. For national surveys such as CCHS, where Aboriginal people are not specifically targeted for inclusion, but who “happen” to be sampled as part of a regional population, sample size tends to be small.

To illustrate these differences, Table II shows the number of Aboriginal people sampled in each cycle of CCHS, APS 200, 2006 and 2012, and RHS 2002/03 and 2008/10.

**Table II:** Number of Aboriginal identity respondents in the Canadian Community Health Surveys, Aboriginal Peoples Survey and First Nations Regional Health Survey

	Territories					
			
Survey	Yukon	NWT	Nunavut	Total	Provinces	Total Canada
CCHS 2000/01	143	507	501	1,151	2,747	3,898
CCHS 2003	149	387	453	989	3,820	4,809
CCHS 2005	163	414	538	1,115	5,201	5,316
CCHS 2007/08	288	615	536	1,439	5,370	6,809
CCHS 2009/10	264	536	461	1,261	5,095	6,356
CCHS 2011/12	260	522	533	1,315	5,215	6,530
APS 2001	1,716	4,912	4,597	11,225	87,424	98,649
APS 2006	919	2,275	3,678	6,872	54,169	61,041
APS 2012	541	1,848	1,609	3,998	34,147	38,145
RHS 2002/03	1,027	1,407	0	2,434	20,168	22,602
RHS 2008/10	1,443	1,562	0	3,005	18,752	21,757

APS 2001 data (aged 0+) from Concepts and Methods, Table 1b, p. 14. APS 2006 (aged 6+) data from Concepts and Methods, Table 1, p. 17. APS 2012 (aged 6+) data from Concepts and Methods, Table 4, p. 26, definition 2. RHS data (aged 0+) from Yukon and NWT regional reports and National reports. CCHS data (aged 12+) computed from masterfiles at the Toronto Region Statistics Canada Research Data Centre. The Aboriginal identity count is based on the responses to question SDCA_7L in CCHS 2000/01, SDCC_7L in CCHS 2003 and SDCE_7L in CCHS 2005: “People in Canada come from many different cultural and racial backgrounds. Are you … Aboriginal Peoples of North America (American Indian, Métis or Inuit)”? In mid-cycle (June 2005), a new question SDC_Q4_1 was added “Are you an Aboriginal person, that is, North American Métis, or Inuit.” The Aboriginal identity count for 2005 is a sum of responses to SDCE_7L and SDC_Q4_1. In CCHS 2007 and thereafter, SDCE_7L was discontinued and the Aboriginal identity count is based on responses to SDC_Q4_1.

It is possible to combine single years for comparison with the earlier pre-2007 bi-annual surveys. Multiple cycles of CCHS can also be merged to increase sample size for specific population subgroups (19). Statistics Canada has merged the 4 annual surveys 2007–2010 into one data set with the specific purpose of enabling comparisons among First Nations, Métis, Inuit and non-Aboriginal people (20). Select indicators from this merged data set are available from CANSIM Tables 105-0512 and 105-0513.

## Survey contents

Health surveys generally focus on 3 broad areas – health status, health determinants and health care. Within each, there may be scores of modules and hundreds of questions covering every conceivable aspect of health. This article deals with health *care* in greater detail, although variables in the other 2 categories can be associated with health care variables. While overall the 3 surveys – CCHS, APS and RHS – tend to have similar contents, differences in the precise wording of the questions can result in variation in prevalence estimates of various conditions and attributes of interest. Compounding the problem are changes over time in terms of definitions, questions and even basic concepts.

Surveys based on interviews by necessity rely on self-reports of health events or issues, be they past health conditions, health-related behaviours or health care use. They suffer from the lack of diagnostic precision and recall bias. This is offset by the advantages of large, nationally/regionally representative samples.

Unless specified otherwise, CCHS refers to the 2007 and later cycles. Differences among them are minor, compared to differences from the earlier pre-2007 cycles. APS refers to the 2001 and 2006 core component, the additional health questions in the Métis and Arctic supplements, and APS 2012 with its extended health supplement. RHS refers to the 2002/03 cycle and the 2008/10 cycle.

### Measures of health status

Table III lists the types of health status measures found in the contents of CCHS, APS and RHS. These are based on questions on self-perceived overall health, history of past chronic diseases, activity limitation, psychological well-being, etc. CCHS has the most extensive battery of questions covering satisfaction with life, coping with stress, self-esteem, distress, depression, etc. At the other extreme is APS, which has no question on mental health in its core questionnaire in 2001 and 2006 (but present in the Métis supplement), a situation that was partially remedied in 2012. Note that the table represents broad concepts or domains and do not imply equivalence in the questions used, although generally they are comparable.

**Table III:** Coverage of measures of health status in selected surveys

		APS			RHS	
			
Content	CCHS	2001	2006	2012	2002/03	2008/10
Self-rated health						
Health utility index						
Activity limitation						
Chronic conditions^a^						
Diabetes						
Arthritis/rheumatism						
Back pain/problem						
Osteoporosis						
Respiratory						
Cardiovascular						
Cancer						
Tuberculosis						
Injuries			M			
Dental health						
Psychological/mental health^b^	O	M	M			
Suicide thoughts/attempts	O	M	M			

CCHS refers to common content in most years, except where indicated (O=optional content, not available from all regions).

a Not all chronic diseases asked are listed in table. Respiratory diseases include chronic bronchitis and emphysema (singly or combined as chronic obstructive pulmonary disease), and asthma; cardiovascular diseases include heart disease, high blood pressure and effects of stroke.

b Broad category includes psychological well-being, personal wellness, depression, distress, stress.

M – present only in Métis supplement of APS 2001 and 2006, not in core questionnaire.

Both CCHS (21–24) and APS (25–27) have been used extensively in studies of the health status of Aboriginal and northern populations.

A history of 1 or more reported chronic diseases can be expected to have a strong impact on the use of health care services. All surveys ask about the history of having been diagnosed with a chronic disease such as diabetes, arthritis, heart disease and chronic respiratory disease. As expected, the proportion of people with no chronic diseases decreases with age, whereas proportionately more people have 2 or more chronic diseases as they grow older (26). The prevalence of several chronic diseases differ between Aboriginal and non-Aboriginal people. Aboriginal people in the North tend to have lower prevalence of chronic diseases than Aboriginal people in the provinces (22). The grouping of First Nations, Inuit and Metis together into a single “Aboriginal” group tends to mask differences in disease risk among these groups (25). The same survey, repeated over time using the same methodology, can demonstrate time trends in the prevalence of a chronic disease (24).

### Measures of health determinants

Many factors or determinants have been identified to contribute to the development or prevention of specific diseases or poor health outcomes. These relate to individual biology, behaviours and the broader physical and social environment. For Aboriginal people, there are additional factors relating to their cultural background and historical experiences. Table IV summarizes the coverage of these topics in the various surveys. As expected, Aboriginal focused surveys such as the APS and RHS but not the CCHS contain questions on the history of having attended residential schools by the respondent or his/her family members and issues such as spirituality and participation in traditional pursuits such as hunting, trapping and fishing. As in Table III and IV conveys only the broad concepts or domains. The 3 surveys (and their different cycles) vary according to the amount of detail and the exact questions used. APS 2012, for example, has very comprehensive coverage of education and employment.

**Table IV:** Coverage of measures of health determinants in selected surveys

		APS			RHS	
			
Content	CCHS	2001	2006	2012	2002/03	2008/10
Socio-economic status						
Education						
Employment						
Income						
Housing						
Food security						
Obesity						
Diet/nutrition						
Fruit and vegetables			M			
Junk foods			M			
“Country” foods		A	AM			
Behaviours						
Physical activity		M	M			
Smoking						
Alcohol use						
Drug use						
Gambling						
Sexual activities						
Residential school attendance						
Social support						
Cultural						
Aboriginal languages spoken						
Hunting/fishing/trapping						
Spirituality						
Community problems						

CCHS refers to common content in most years, except where indicated (O=optional content, not available from all regions). “A” and “M” indicate the question was only asked in the Arctic and Métis supplements.

**Fig. 1:**
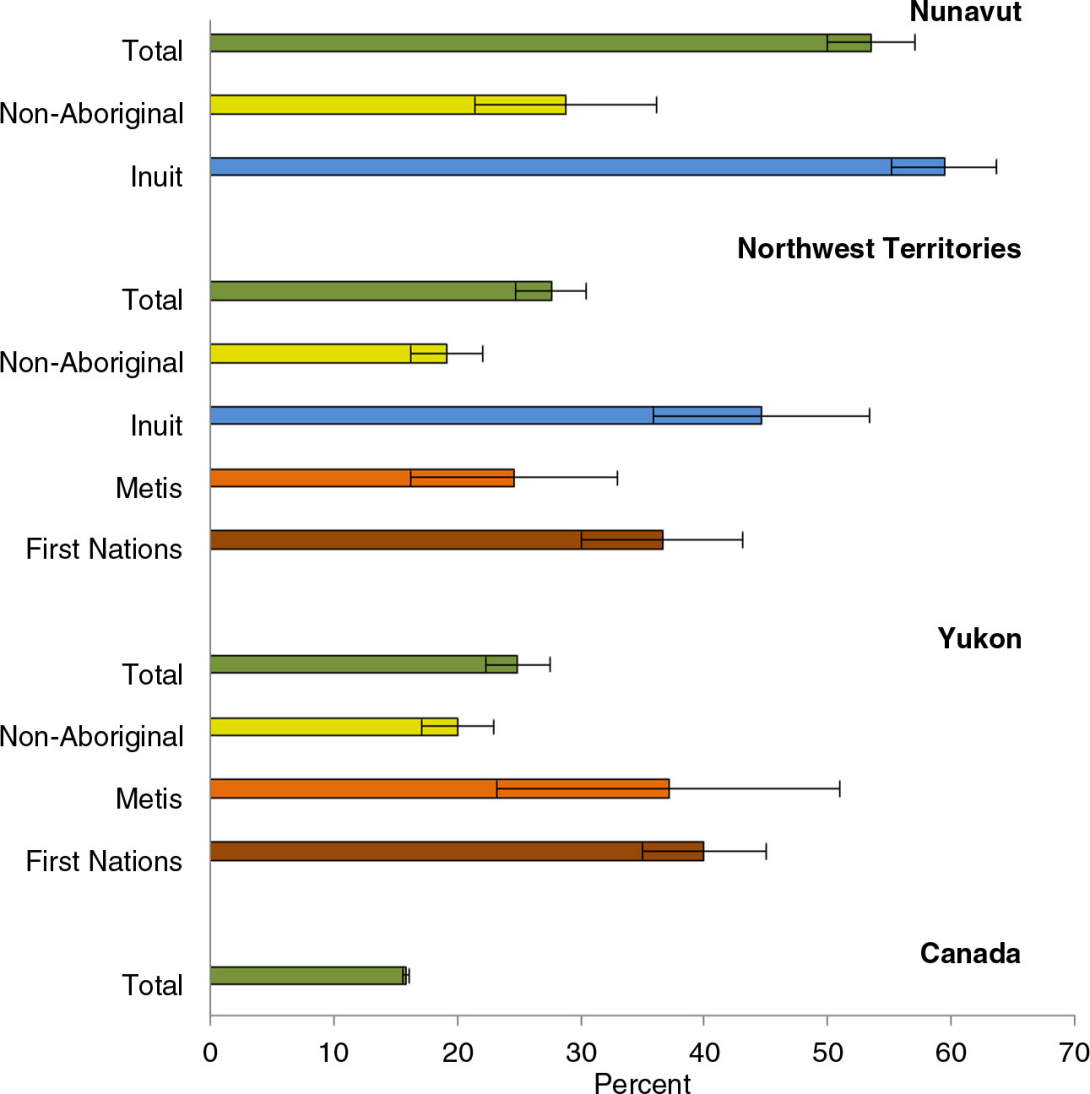
Age-standardized prevalence of daily smoking in the northern territories and Canada. Source: CCHS 2007–2010, as reported in CANSIM Table 105-0513. Population aged 12+. Error bars refer to 95% confidence intervals. Age-standardized to the 1991 Canadian population.

As an illustration of the substantial variation in distribution of risk factors between North and South, and within the North, among different Aboriginal groups, the prevalence of daily smoking is shown in Fig. 1, based on the merged data sets of CCHS 2007–2010.

### Measures of health care

The types of health care issues investigated by CCHS, APS and RHS are limited to access to care, use of different types of services, patient satisfaction and participation in preventive practices (Table V). It can be seen that many health care issues are not adequately covered by surveys in the North.

**Table V:** Coverage of measures of health care in selected surveys

	CCHS 2000/01			CCHS 2003			CCHS 2005			CCHS 2007/08			CCHS 2009/10			CCHS 2011/12			APS			RHS	
								
Indicator	YT	NT	NU	YT	NT	NU	YT	NT	NU	YT	NT	NU	YT	NT	NU	YT	NT	NU	01	06	12	02/03	08/10
Health care access/availability																							
Routine regular physician care																			M	M			
Difficulty accessing specialist services																							
Insurance coverage for glasses/dental care																							
Difficulty accessing NIHB services																							
Unmet health care needs														2010					M				
Wait-time for surgical/diagnostic services																							
Home care needed but not received																							
Continuity of primary care providers																							
Affordability of prescription drugs																			M	M			
Health services utilization																							
Contact with health professionals														2010									
Stay in hospital/nursing home														2011					M				
Contact with mental health professional																							
Dental visits																			M				
Diabetes care																							
Type of home care received																							
Use of preventive services																							
Blood pressure check																			M	M			
Blood test																							
Test for diabetes																			M	M			
Test for cholesterol																							
Flu shots																							
Colorectal cancer screening																							
Mammography																			M	M			
Pap smear test																			M	M			
Prostate cancer screening																				M			
Physical check-up											2008								M				
Spirometry											2008						2012						
Breast examinations by health professional																			M				
Breast self-exam																							
Eye examinations																							
Smoking cessation counselling by physician																							
Patient satisfaction																							
Rating of quality and satisfaction																				M			
Specific to community-based care																							
Satisfaction with physician availability																				M			
Traditional Aboriginal medicine																							
Use of traditional healer/medicine																							
Availability in community																							
Difficulty access																							

Year in a cell indicates that the question was included in 1 year in the 2-year period. M=included only Métis supplement; NIHB=Non-Insured Health Benefits program administered by Health Canada for First Nations people.

Under health care access and availability are questions on whether one has a regular medical doctor, where the care is obtained, and the reasons for not having one; difficulties accessing specialists and elective surgery and the reasons why; the type of health services that are needed but not received; and the waiting time for surgical and diagnostic services and the impact of the waiting on the person's health. There are questions on insurance coverage for eye glasses, dental care and services not covered by universal health care plans in CCHS. As these services are generally available free of charge to First Nations people under Health Canada's Non-Insured Health Benefits program (NIHB), RHS instead asks about difficulties in accessing these services. Asked in RHS 2008/10 but not in either CCHS or APS is the continuity of primary care services – how many times the providers have changed in the past 12 months. The Métis supplement of APS 2001 and 2006 ask if the respondent could not fill a prescription because of the lack of money. This issue is relevant to Métis who do not have access to NIHB, unlike First Nations patients.

Several questions focus on actual use in the past 12 months of specific types of health services such as hospital, home care, dental care and mental health care; and contact with specific types of health professionals such as physicians, nurses and dentists. The list of health professionals varies among different surveys. Given the high prevalence of diabetes in the First Nations population, the questions on diabetes care in RHS are more detailed than those in CCHS, including visits to diabetes clinic and access to diabetes education.

Questions on preventive services focus on ever used, last time used and reasons for not using. Indices can be created to assess adherence to current guidelines such as target age group and frequency of testing. As can be seen from Table V, the core APS has few questions on preventive practices. However, the Métis supplements in 2001 and 2006 did contain several related questions.

Respondents are asked to rate (excellent, very good, etc.) the quality of the services they received (any health service, hospital, physicians) and if they are satisfied. CCHS 2005 added questions specific to community-based health care. The optional module on satisfaction with the availability of physicians was not selected by the territories, perhaps in recognition of the absence of physicians located in small, remote communities. However, it was included in the Métis supplement of APS 2006.

As expected, APS and RHS have an interest in the use and availability of traditional Aboriginal healers and medicine. Data suggest that Aboriginal people in the North tend to use traditional healers less than those living in the provinces (26).

Absent from the list are important indicators such as comprehensiveness and scope, coordination and teamwork, cultural sensitivity, patient empowerment, communication and support for self-management, etc. Conspicuous in their absence are specific references to rehabilitative services, an important third pillar to curative and preventive services.

### North–South differences in health care

There are substantial differences between North and South, and between Aboriginal and non-Aboriginal people within the North in terms of health care use. Aboriginal people use physicians much less, and nurses much more often, than non-Aboriginal people in the North (21), or Aboriginal people in the South (25,26). This reflects the nurse-based primary care system in remote communities in the North.

**Fig. 2:**
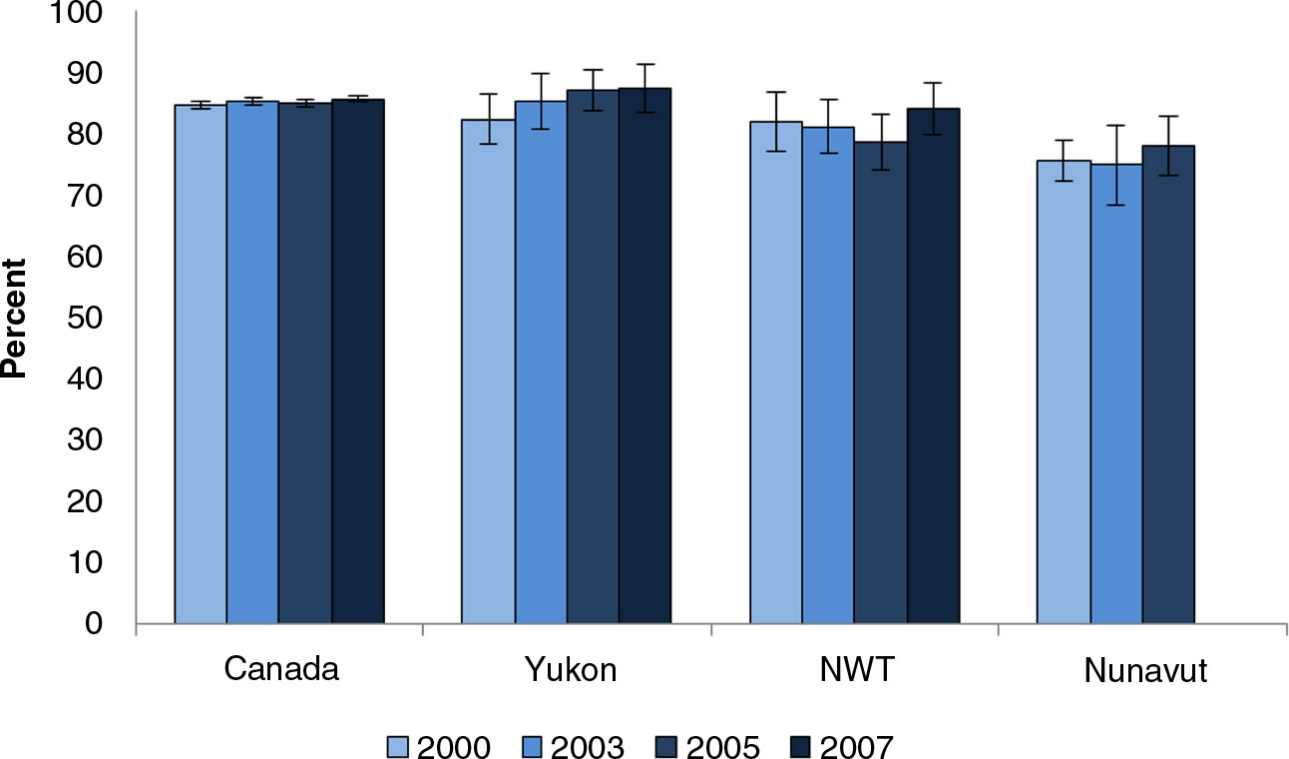
Proportion of population who are very and somewhat satisfied with health care services they received past 12 months. Source: CCHS 2000, 2003, 2005 and 2007, as reported in CANSIM Table 105-4080. Question not asked in Nunavut in 2007.

In terms of patient satisfaction with the health services they receive, there is no significant difference between Yukon, the NWT and the whole of Canada in the proportion who were very and somewhat satisfied, as all are in the 80%+ range (Fig. 2). However, the proportion in Nunavut is significantly lower.

**Fig. 3:**
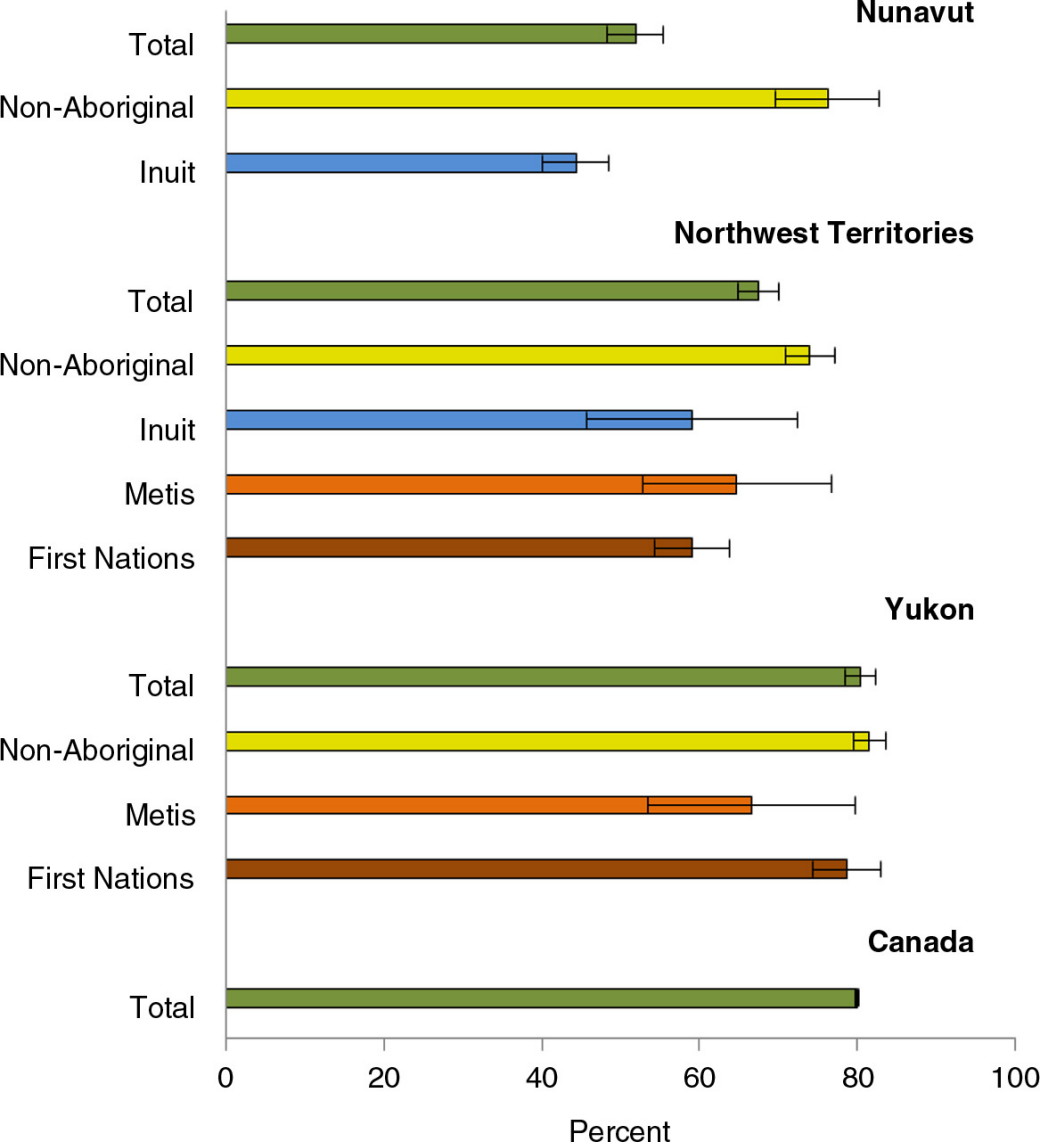
Proportion of population who reported having consulted a medical doctor past 12 months. Source: CCHS 2007–2010, as reported in CANSIM Table 105-0513. Population aged 12+. Error bars refer to 95% confidence intervals. Medical doctors include family doctors/general practitioners and specialists. Crude prevalence, rather than age-standardized prevalence, is shown as it reflects the actual health care use by the population, and not a hypothetical standardized population.

It should be recognized that while residents of the territories have less health care access than Canadians in the south, non-Aboriginal people in the North, who are concentrated in the territorial capital cities, in fact differ little from other Canadians. Lumping all Aboriginal groups together can also be misleading, as there are substantial differences among them as well. This is evident from Fig. 3, which compares the use of physician services. Non-Aboriginal people in the territories basically do not differ from Canadians nationally. Inuit in Nunavut have the lowest, while Yukon First Nations have the highest prevalence of use.

It has been shown in a study using APS 2001 and CCHS 2000/01 that the determinants or correlates of physician use differ between Aboriginal and non-Aboriginal people. Thus, household income was a significant factor (i.e. low income, low usage) in the non-Aboriginal but not the Aboriginal population, whereas household size (i.e. more crowding, higher usage) was a significant factor in the Aboriginal but not the non-Aboriginal population (27). However, the study involved the whole of Canada and did not look at the north specifically.

The North actually does relatively well in certain preventive services based on some surveys. For example, Pap smear coverage is high, with little difference between Aboriginal and non-Aboriginal people. Participation in mammography, however, is substantially lower, perhaps a reflection of the lack of access to facilities with the equipment for residents of remote communities (28).

Health care cannot be assessed by asking questions only of users. Providers, managers and policymakers need also to be surveyed. System-wide characteristics such as effectiveness and efficiency, governance and stewardship, infrastructure and workforce cannot be determined from individual-based surveys but require reviews of other data systems such as health care administrative databases. The Canadian Institute for Health Information is a rich resource for such databases for Canada and its provinces and territories.

## Conclusions and recommendations

Three surveys – CCHS, APS and RHS – conducted since 2000 provide some information relevant to health care, with sufficient sample size to permit separate estimates for the North and its Aboriginal populations.

Existing data are insufficient in assessing health care beyond very basic indicators relating to access to certain services, contact with different categories of health professionals, unmet health needs and use of preventive services.

**Fig. 4:**
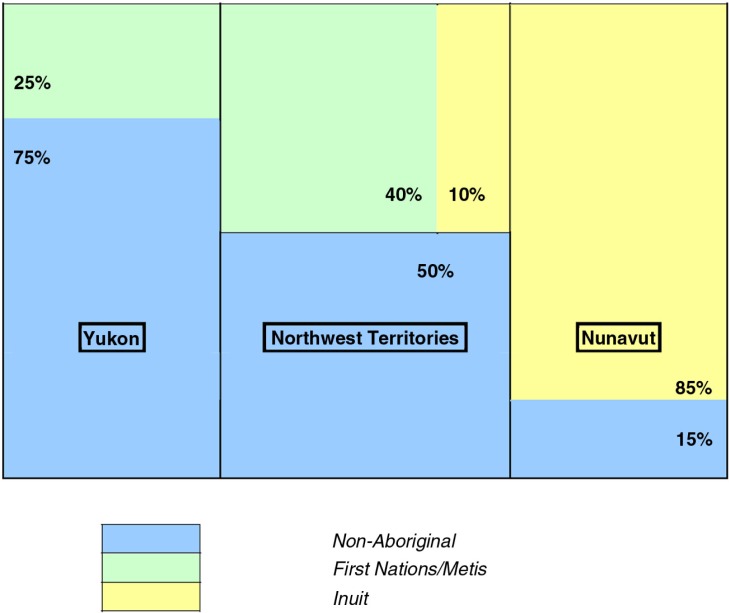
Population distribution of Aboriginal people in the 3 territories. Source: 2006 Census.

Disparities in certain health care indicators between North and South are apparent – in general, Yukon tends to be closest to Canadian norm, Nunavut furthest from, with NWT somewhere in between. This is very much a reflection of the demographic make-up of the 3 territories, with Aboriginal people accounting for about 25% of the population of Yukon, 50% of the NWT and 85% of Nunavut (Fig. 4). Substantial differences also exist among the territories with regard to socio-economic status.

Health care for non-Aboriginal residents in the North is not very different from most Canadians. Non-aboriginal residents in the North tend to live in the territorial capitals, where health care facilities are not very different from similar-sized cities in Canada. Thus, 67% of Yukon's population live in Whitehorse, of whom 81% are non-Aboriginal; 45% of the population of the NWT live in Yellowknife, of whom 78% are non-Aboriginal; while only 20% of Nunavut's population live in Iqaluit, of whom 40% are non-Aboriginal (29).

Aggregating all First Nations, Inuit and Métis into a single Aboriginal group obscures important differences among them in terms of health status, health determinants and health care.

The Introduction of this article states that a stand-alone survey focused on health care in the North is resource intensive and time consuming. Given the patchiness of coverage of even basic indicators in the North and its subpopulations across surveys, a comprehensive survey of health care is needed. Such a survey needs to be a sufficient sample size to enable disaggregation of the sample into urban and remote, Aboriginal and non-Aboriginal, and First Nations, Inuit and Métis within the North. Analytical studies can also be conducted to investigate the correlations and interactions among health status, health determinants and health care and assess whether such relationships differ among the different population groups. Such a survey can also focus on individuals with complex health needs, which can be identified as those with multiple chronic disease co-morbidities, disabilities, and mental health problems.

## References

[1] Young TK, Chatwood S (2011). Health care in the North: what Canada can learn from its circumpolar neighbours. Can Med Assoc J.

[2] Young TK, Marchildon G (2012). A comparative review of circumpolar health systems. Circumpolar Health Supplements.

[3] Young TK, Chatwood S, Bjerregaard P (2012). Global health – a circumpolar perspective. Am J Public Health.

[4] Young TK (2012). Circumpolar health atlas.

[5] Young TK, Bjerregaard P (2008). Health transitions in Arctic populations.

[6] Swain L, Catlin G, Beaudet MP (1999). The National Population Health Survey – its longitudinal nature. Health Rep.

[7] Béland Y (2002). Canadian community health survey – methodological overview. Health Rep.

[8] Poppel B (2015). SLiCA: Arctic living conditions.

[9] Statistics Canada (2003). Health Services Access Survey. http://www23.statcan.gc.ca/imdb-bmdi/document/3226_D14_T9_V1-eng.pdf.

[10] Canadian Institute of Health Information (2009). Experiences with primary health care in Canada. Analysis in Brief.

[11] Canadian Institute of Health Information (2012). Disparities in primary health care experiences among Canadians with ambulatory care sensitive conditions. Analysis in Brief.

[12] Tremblay M, Wolfson M, Connor Gorber S (2007). Canadian Health Measures Survey: rationale, background and overview. Health Rep.

[13] Saudny H, Leggee D, Egeland G (2012). Design and methods of the adult Inuit Health Survey 2007–2008. Int J Circumpolar Health.

[14] Commonwealth Fund International health policy surveys.

[15] Statistics Canada (2007). How statistics Canada identifies Aboriginal people.

[16] Chan WW, Ng C, Young TK (2013). How we identify and count Aboriginal people – does it make a difference in estimating their disease burden?. Chronic Dis Can.

[17] Statistics Canada (2015). National Household Survey, 2011: sampling and weighting technical report.

[18] Harvard Project on American Indian Economic Development (2006). Review of the First Nations Regional Longitudinal Health Survey (RHS) 2002/03.

[19] Thomas S, Wannell B (2009). Combining cycles of the Canadian Community Health Survey. Health Rep.

[20] Gionet L, Roshanafshar S (2013). Select health indicators of First Nations people living off reserve, Métis and Inuit.

[21] Tjepkema M (2002). The health of the off-reserve Aboriginal population. Health Rep.

[22] Lix LM, Bruce S, Sarkar J, Young TK (2009). Risk factors and chronic conditions among Aboriginal and non-Aboriginal populations. Health Rep.

[23] Deering KN, Lix LM, Bruce S, Young TK (2009). Chronic diseases and risk factors in Canada's northern populations: longitudinal and geographic comparisons. Can J Publ Health.

[24] Sarkar J, Lix L, Bruce S, Young TK (2010). Ethnic and regional differences in prevalence and correlates of chronic diseases and risk factors in northern Canada. Prev Chronic Dis.

[25] Ng C, Chatwood S, Young TK (2010). Arthritis in the Canadian Aboriginal population: north-south differences in prevalence and correlates. Chron Dis Can.

[26] Wilson K, Rosenberg MW, Abonyi S (2011). Aboriginal peoples, health and healing approaches: the effects of age and place on health. Soc Sci Med.

[27] Wilson K, Rosenberg MW, Abonyi S, Lovelace R (2010). Aging and health: an examination of differences between older Aboriginal and non-Aboriginal people. Can J Aging.

[28] McDonald JT, Trenholm R (2010). Cancer-related health behaviours and health service use among Inuit and other residents of Canada's north. Soc Sci Med.

[29] Statistics Canada 2006 census community profiles.

